# The Profile of Non-Communicable Disease (NCD) research in the Middle East and North Africa (MENA) region: Analyzing the NCD burden, research outputs and international research collaboration

**DOI:** 10.1371/journal.pone.0232077

**Published:** 2020-04-27

**Authors:** Ajay Aggarwal, Preeti Patel, Grant Lewison, Abdulkarim Ekzayez, Adam Coutts, Fouad M. Fouad, Omar Shamieh, Rita Giacaman, Tezer Kutluk, Rima Abdul Khalek, Mark Lawler, Peter Boyle, Diana Sarfati, Richard Sullivan

**Affiliations:** 1 Institute of Cancer Policy, Cancer Epidemiology, Population & Global Health, School of Cancer Sciences, King’s College London, London, United Kingdom; 2 Department of Clinical Oncology, Guy’s & St.Thomas’ NHS Trust, London, United Kingdom; 3 Department of War Studies, King’s College London, London, United Kingdom; 4 Conflict and Health Research Group, King’s College London, London, United Kingdom; 5 Department of Sociology, University of Cambridge, Cambridge, United Kingdom; 6 Global Health Institute/Department of Epidemiology and Population Health, American University of Beirut, Beirut, Lebanon; 7 King Hussein Cancer Center, Amman, Jordan; 8 Institute of Community and Public Health, Birzeit University, Birzeit, West Bank, occupied Palestinian territory; 9 Department of Pediatric Oncology, Hacettepe University Faculty of Medicine, Ankara, Turkey; 10 Queen’s University Belfast, Centre for Cancer Research and Cell Biology, Belfast, United Kingdom; 11 International Prevention Research Institute, Lyon, France; 12 Department of Public Health, University of Otago, Wellington, New Zealand; University of Central Florida, UNITED STATES

## Abstract

**Objectives:**

Despite the rising risk factor exposure and non-communicable disease (NCD) mortality across the Middle East and the North African (MENA) region, public health policy responses have been slow and appear discordant with the social, economic and political circumstances in each country. Good health policy and outcomes are intimately linked to a research-active culture, particularly in NCD. In this study we present the results of a comprehensive analysis of NCD research with particular a focus on cancer, diabetes and cardiovascular disease in 10 key countries that represent a spectrum across MENA between 1991 and 2018.

**Methods:**

The study uses a well validated bibliometric approach to undertake a quantitative analysis of research output in the ten leading countries in biomedical research in the MENA region on the basis of articles and reviews in the Web of Science database. We used filters for each of the three NCDs and biomedical research to identify relevant papers in the WoS. The countries selected for the analyses were based on the volume of research outputs during the period of analysis and stability, included Egypt, Iran, Jordan, Kuwait, Lebanon, Oman, Qatar, Saudi Arabia, Turkey and the United Arab Emirates.

**Results:**

A total of 495,108 biomedical papers were found in 12,341 journals for the ten MENA countries (here we consider Turkey in the context of MENA). For all three NCDs, Turkey's output is consistently the highest. Iran has had considerable growth in research output to occupy second place across all three NCDs. It appears that, relative to their wealth (measured by GDP), some MENA countries, particularly Oman, Qatar, Kuwait and the United Arab Emirates, are substantially under-investing in biomedical research. In terms of investment on particular NCDs, we note the relatively greater commitment on cancer research compared with diabetes or cardiovascular disease in most MENA countries, despite cardiovascular disease causing the greatest health-related burden. When considering the citation impact of research outputs, there have been marked rises in citation scores in Qatar, Lebanon, United Arab Emirates and Oman. However, Turkey, which has the largest biomedical research output in the Middle East has the lowest citation scores overall. The level of intra-regional collaboration in NCD research is highly variable. Saudi Arabia and Egypt are the dominant research collaborators across the MENA region. However, Turkey and Iran, which are amongst the leading research-active countries in the area, show little evidence of collaboration. With respect to international collaboration, the United States and United Kingdom are the dominant research partners across the region followed by Germany and France.

**Conclusion:**

The increase in research activity in NCDs across the MENA region countries during the time period of analysis may signal both an increasing focus on NCDs which reflects general global trends, and greater investment in research in some countries. However, there are several risks to the sustainability of these improvements that have been identified in particular countries within the region. For example, a lack of suitably trained researchers, low political commitment and poor financial support, and minimal international collaboration which is essential for wider global impact.

## Introduction

The social, economic and political impacts of non-communicable diseases (NCDs) are now recognized to be some of the greatest challenges facing countries in their efforts to deliver on Sustainable Development Goals (SDGs) and Universal Health Coverage (UHC) [1]. Whilst the public policy dialogue has focused on communicable diseases, research into NCDs has received little attention [2] [3].

Relative to their available economic resources, many of the Middle East and North African (MENA) countries have not, historically, achieved good health outcomes [4]. The recent *Health Systems Reform* series on health in the MENA region provided evidence of the heterogeneous patterns of health outcomes across this region [5]. Despite poor outcomes in conflict-impacted countries such as Syria, Iraq, Libya, Yemen and Afghanistan over the last two decades, child and adult mortality rates have declined across the MENA region as a whole, particularly in Saudi Arabia, Iran, Qatar, the United Arab Emirates, Oman, and Turkey [6, 7]. [8].

The MENA population most at risk of NCDs, i.e. those aged over 60, currently ranges from 0.8% in the UAE to 10.6% in Turkey [9]. These percentages are expected to increase significantly by 2030 as a result of the demographic transition due to continuing improvements in life expectancy. Compounding this demographic effect, is exposure to NCDs risk factors such as tobacco, obesity and hypertension, which is highly prevalent across the MENA region.

In Bahrain, Egypt, Jordan, Kuwait, Saudi Arabia and the United Arab Emirates, adult obesity (BMI ≥ 30) is becoming a major issue in both men and women [10], and almost 25% of the MENA population suffers from hypertension [11]. Estimates from the Global Burden of Disease (GBD) study show that the highest prevalence of obesity among adults 20 years or older in 2015 was observed in Qatar, with 42.5% among males and 52.4% among females, and in the younger population of children 2–19 years in Kuwait with 22.1% among males and 19.2% among females (GBD 2015).

Whilst tobacco use is declining worldwide, according to the World Health Organization (WHO) Global Report on Trends in Tobacco Smoking 2000–2025, the prevalence of tobacco smoking in the MENA region is projected to increase between 2010 and 2025 [12].

There is also wide variation in the demographic patterns of cigarette smoking, with prevalence among older men at 30–50% in Egypt, Jordan and Bahrain, compared to 7–15% in the UAE and Oman [13]. Moreover, water-pipe tobacco smoking (e.g., hookah, shisha, arghile, narguileh) is increasing among the youth in the region.

Cardiovascular disease, cancer and diabetes represent up to one third of the current disease burden in the MENA region. The cardiovascular disease burden has remained relatively stable between 2000 and 2015 rising from 17.5% of all DALYS to 18.9%. The burden is twice that of Cancer, the burden of which has increased from 8.6% to 10.2% of all DALYs in the same time period. Diabetes represented 4.3% of all DALYs in 2015, up from 2.5% in 2000.

Despite the rising risk factor exposure and NCD mortality, regional public health policy responses have been slow. Historical and future R&D activity in NCD is foundationally dependent on the knowledge economy of these countries, however, in many places this is still lagging behind (https://gulfbusiness.com/the-middle-easts-state-of-knowledge/). In the face of the significant public health threat from NCDs and the drive to improve health systems research and address the knowledge gap in the MENA region, there is a need for evidence-based inter-sectoral measures for NCD prevention and care in the MENA region and for an understanding of how to improve the NCD research capacity and capability across the region.

To achieve this, it is important to understand from a public policy perspective how, why, and which particular NCD research domains have evolved. For example, how do different countries influence the NCD research agenda, either through the volume of research they publish, the citation impact of their articles, or their commitment to particular research domains (eg, basic science)? An empirical analysis of research outputs would also highlight gaps and provide direction as to which research areas should be prioritized to meet current and future challenges.

Using a bibliometric approach, we present a high resolution analysis of NCD research in ten key “Bell weather” countries that reflect the (stable, permissive) diversity of the MENA region between 1991 and 2018 (28 years) to characterize its breadth and depth and how these have evolved over the course of nearly three decades. This type of analysis is now used routinely in public policy analysis to study research domains [14–17]. A bibliometric approach allows one to understand and characterise research in the region, to provide an understanding of the research capacity to manage this burden (workforce and time required to produce research outputs) relative to wealth and individual disease burden. In addition, it provides a focus for understanding areas for informing policy by providing insight into research prioritisation, research impact and the extent of regional and international collaborations.

We therefore examine the growth in NCD research outputs over time, the volume of research produced relative to the countries' wealth, population size, and NCD disease burden. In addition, we assess the citation impact of each country’s NCD research as well as the impact of intraregional and international collaborations on the volume and quality of research outputs.

## Methods

A number of different definitions for country composition of the MENA region currently exist [18] with some including Turkey. Our analysis of NCD research activity covers the 10 major research-active countries in this region (defined according to the volume of research outputs) for the 28-year period between 1991 and 2018. The selected countries are: Egypt, Iran, Jordan, Kuwait, Lebanon, Oman, Qatar, Saudi Arabia, Turkey, and the United Arab Emirates. International Standards Organization (ISO) digraph codes for each country are listed in **Table 1**. We excluded countries that are considered politically unstable e.g. Iraq, Yemen and Syria and did not include smaller research producing countries such as Bahrain, Libya, Palestine, as they would not be informative for understanding overall NCD and workforce policy in the region. We also wished to look at broad trends of countries within a specific research-political nexus, hence exclusion of both low output countries and specific countries in North Africa that are not part of the intra-regional research nexus.

**Table 1:** List of 10 countries from MENA region that were the focus of the study.

*Country*	*ISO*	*Country*	*ISO*
Egypt	EG	Oman	OM
Iran	IR	Qatar	QA
Jordan	JO	Saudi Arabia	SA
Kuwait	KW	Turkey	TR
Lebanon	LB	United Arab Emirates	AE

In addition, for each country, we cover the three most prevalent NCDs in the MENA region: cancer, cardiovascular disease and diabetes.

Research papers (articles and reviews) from the 10 selected countries were identified from the Web of Science (WoS) in the selected NCDs by means of three separate algorithms (filters), developed by GL in collaboration with experts in these NCD fields (See Appendix 1). Each of these three filters consisted of two parts: a list of specialist journals for each NCD and a list of specific title words. There were 185 specialist journals listed for cancer, 115 for cardiovascular disease and 36 for diabetes, and there were 323 title words or phrases used for cancer, 125 for cardiovascular disease and 35 for diabetes. Of note the cardiovascular filter includes cerebrovascular accident (CVA). Each filter was independently applied to all papers identified from each of the 10 countries within the WoS. Papers satisfying either criterion (i.e. containing a specific NCD title word or published in an NCD specialist journal), or both, were selected for analysis.

Each of the filters has undergone a process of calibration during the development phase to assess their precision (specificity) and recall (sensitivity)[19]. The filters have been used previously in other analyses[14, 20, 21] and the process of development of the cancer filter for example is listed [22]. For the three NCD domains sensitivity and specificity rates were calculated as follow, with sensitivities and specificities above 90% considered high:

- CARDI p = 0.95, r = 0.90,
- DIABE p = 0.90, r = 0.98
- ONCOL p = 0.95, r = 0.98

A separate filter for biomedical research was developed and also applied to the WoS [19]. The rationale for this was to provide a baseline from which the relative increases in volume of research outputs in the three NCD domains can be considered. It also provides the relevant context for understanding the commitment of each country across all biomedical research domains.

This was based, not on title words, but on words in the addresses of the papers that indicated the name of the department, *e*.*g*. Department of Cardiology, or the institution, such as NIH (US National Institutes of Health). There were a total of 172 terms in this filter, and it has been found to distinguish well between biomedical and non-biomedical papers in multi-disciplinary journals such as *Nature* and *Science*.

### Assessment of research output relative to GDP and disease burden

An analysis was undertaken to determine whether the amount of biomedical research in each MENA country in 2011–18 was commensurate with its overall wealth as measured by Gross Domestic Product (GDP), and also with its respective population size (both for 2014).

The research output in each of the three NCDs was calculated as a proportion of total biomedical research output. A comparison was also made with the relative burden of disease, measured in Disability Adjusted Life-Years (DALYs) attributable to each NCD. Disability Adjusted Life Years are the sum of years of potential life lost due to premature mortality (compared with life expectancy in Japan) and the years of productive life lost due to disability (e.g. pain, loss of vision) based on how bad particular problems are.

These were calculated as percentages of DALYs attributable to all causes for each country in the years 1990 to 2015 [23]. It was therefore possible to compare for each country the proportion of research into each NCD domain relative to its total biomedical research output with the proportion of DALYs attributable to each NCD. This was calculated for two 10-year time periods (1996–2005 compared with DALYs in 2000 and 2009–2018, compared with DALYs in 2010) to assess what the changes in commitment had been relative to the changing disease burden.

### Contribution of basic compared with clinical research over time

The research level of the papers (RLp) was assessed for the papers in each domain, from each country and for the two decades 1996–2005 and 2009–18. The mean research levels give an indication of the countries' commitment to clinical research relative to basic science within that NCD. It is based on the presence of selected “clinical” and “basic science” words in their titles. Groups of papers were assigned a value of RLp between 1.0 (clinical observation) and 4.0 (basic research) depending on whether their titles contained one or more of a list of “clinical words” *e*.*g*., *diagnosis*, *elderly*, “basic words” *e*.*g*. *activation*, *binding*, *chromosome*, or both [24].

### Impact of international collaboration on quantity and impact of research

International collaboration was assessed by calculation of the proportion of biomedical research outputs within each of the MENA countries that had foreign addresses (a proxy for international co-authorship) between 2011 and 2018. For this purpose, the numbers of each country's papers with no other country present among the addresses were determined, and then subtracted from the total output. A further analysis revealed the extent of international biomedical research collaboration between the selected 10 MENA countries and others in the MENA region as well as with the top 10 biomedical research-active countries globally. The indicator used was the Salton Index (SI). This is defined as the ratio of the numbers of joint publications to the square root of the product of the two individual totals, multiplied by 100.

For the biomedical research papers, the Actual Citation Impact (ACI) was calculated as the number of citations received by a paper in the five years beginning in the year of publication. A five-year window was used as it represents a compromise between the need for immediacy (*i*.*e*., citations to recent papers) and stability (*i*.*e*., inclusion of the peak year for citations, usually the second or third year after publication). For the biomedical papers from 2001–2010, ACI values were obtained from the WoS for each of the ten countries and for each publication year. Because these scores varied from year to year, particularly for the smaller countries, three-year running means were calculated so as to smooth out this variation.

## Results

### Outputs of research papers in the three NCDs

**Between 1991–2018,** 495,108 biomedical papers were found in 12,341 journals for the 10 MENA countries. Altogether, in cancer there were 52,911 papers in 4344 journals; in cardiovascular disease, there were 49,234 papers in 4382 different journals, and in diabetes there were 13,298 papers in 2403 journals. Nearly all of the papers were in English (98.5%), but others were in 19 different languages, led by Turkish with 1.2%.

In 2015, 17% of the diabetes papers, 18% of the oncology papers and 21% of the cardiology papers were published in journals from eight of the selected MENA countries (all except for Lebanon and Oman). The papers were primarily from Turkey (61% of the MENA total) and Iran (30%): Saudi Arabia and the UAE contributed less than 4% of the MENA total. The four leading MENA journals are all Turkish: *Archives of the Turkish Society of Cardiology*, *Journal of Clinical and Analytical Medicine*, *Anatolian Journal of Cardiology*, and *Turkish Journal of Thoracic and Cardiovascular Surgery* (all with between 90 and 110 papers).

The research outputs of the 10 MENA countries in the three NCDs for the years 1991–2018 are shown in **S1, S2 and S3 Figs** for cancer, cardiovascular research and diabetes respectively. With few exceptions, there is a trend of increasing outputs over time in all countries for all three NCDs, and Turkey's (TR) output is consistently the highest. Iran (IR) has overtaken several other countries to occupy second place across all three NCDs. Egypt's (EG) output has also expanded quite quickly to overtake that of Saudi Arabia (SA).

### The correlation between country biomedical research outputs and wealth, population and disease burden

**Fig 1:**
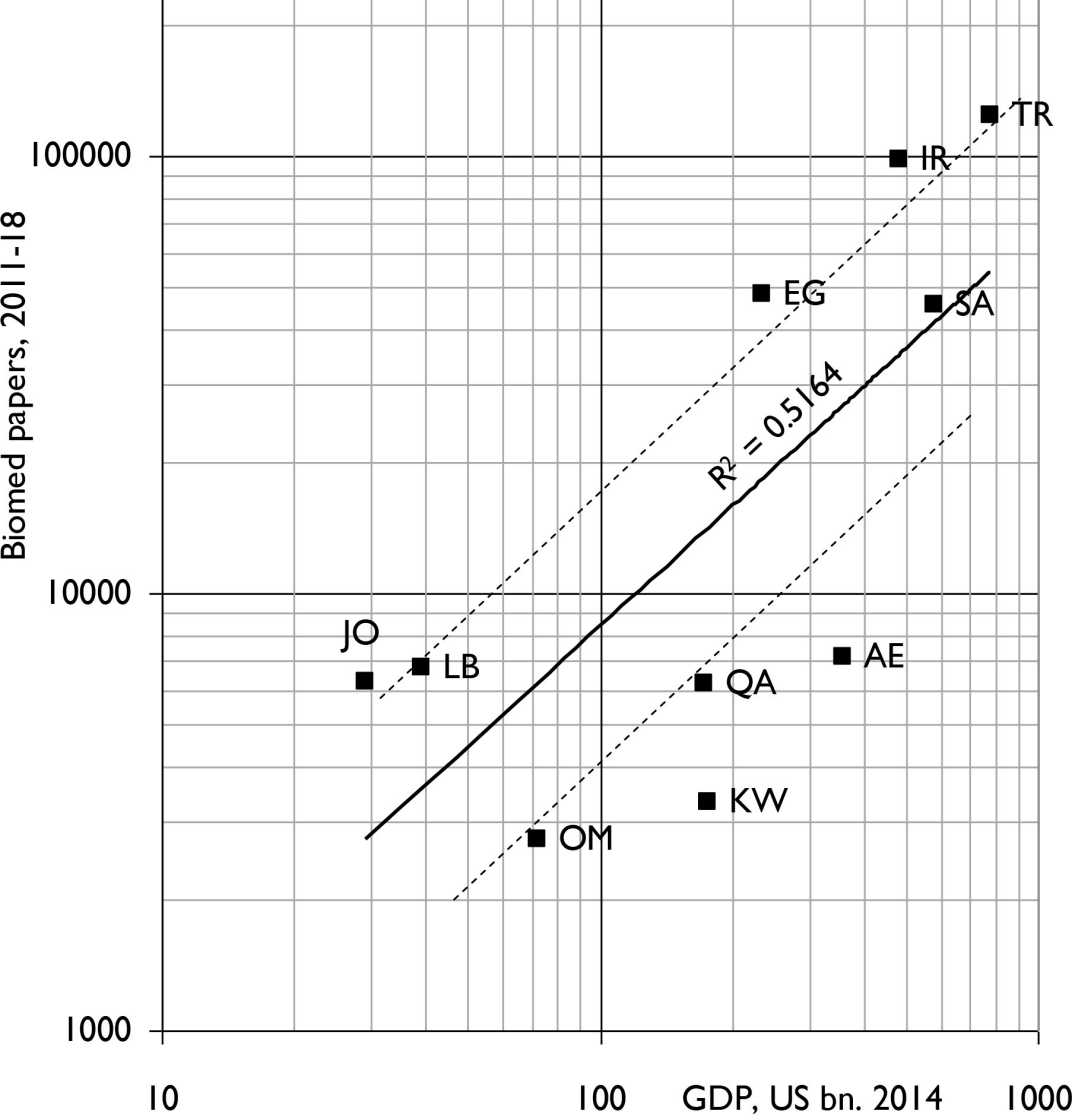
Biomedical research outputs, 2011–18, from the ten IW countries (integer counts) compared with their gross domestic products in 2014. Solid line is best fit to data for the 10 countries on least-squares basis. Dashed lines represent outputs half, and twice those expected from countries' GDP. For country codes, see Table 1.

**Fig 2:**
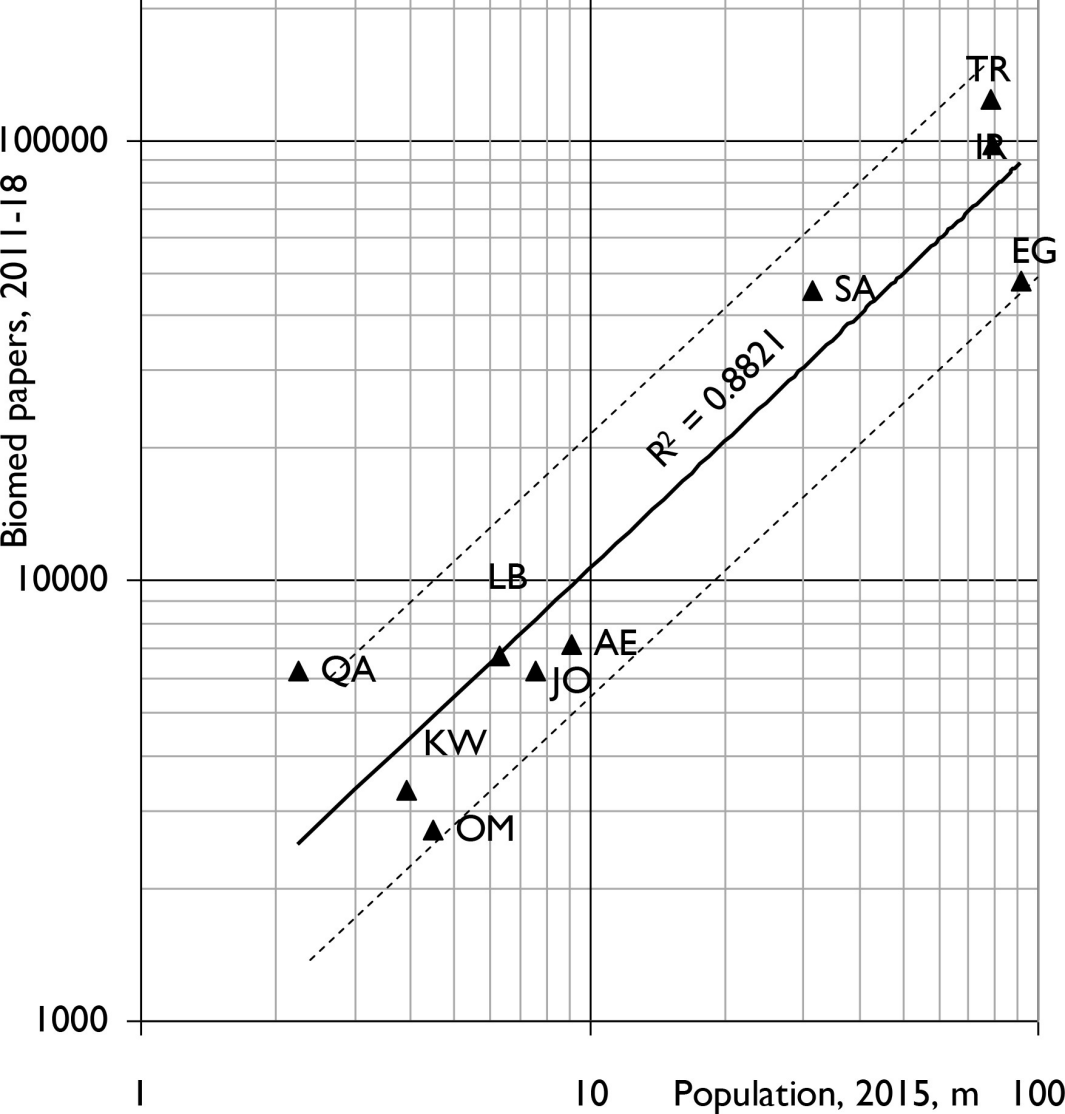
Biomedical research outputs, 2011–18, from the ten IW countries (integer counts) compared with their populations in 2014. Solid line is best fit to data for the 10 countries on least-squares basis. Dashed lines represent outputs half, and twice those expected from countries' populations. For country codes, see Table 1.

During the eight years, 2011–18, the 10 MENA countries published 328,585 biomedical papers (5.4% of the world total) in 10,855 different journals. Biomedical research activity was poorly correlated (r^2^ = 0.52) with the wealth of the 10 MENA countries in 2014 **(Fig 1)** but much better with their populations (r^2^ = 0.88) **(Fig 2)**. In both Figs 1 and 2, the spots for Kuwait (KW), Oman (OM) and the United Arab Emirates (AE) all lie below the regression-line, and those for Iran (IR) and Turkey (TR) lie above the line.

The relationship between disease burden and research activity is complex, with a heterogeneous picture across the 10 MENA countries both within each NCD and between NCDs. With a few exceptions, the MENA countries do more research on cancer than the burden (an average of 6.3% of all DALYs) would appear to justify. Conversely cardiovascular disease (with 15.5% of all DALYs) and diabetes (with 2.9%) appear to be under-researched.

**Fig 3:**
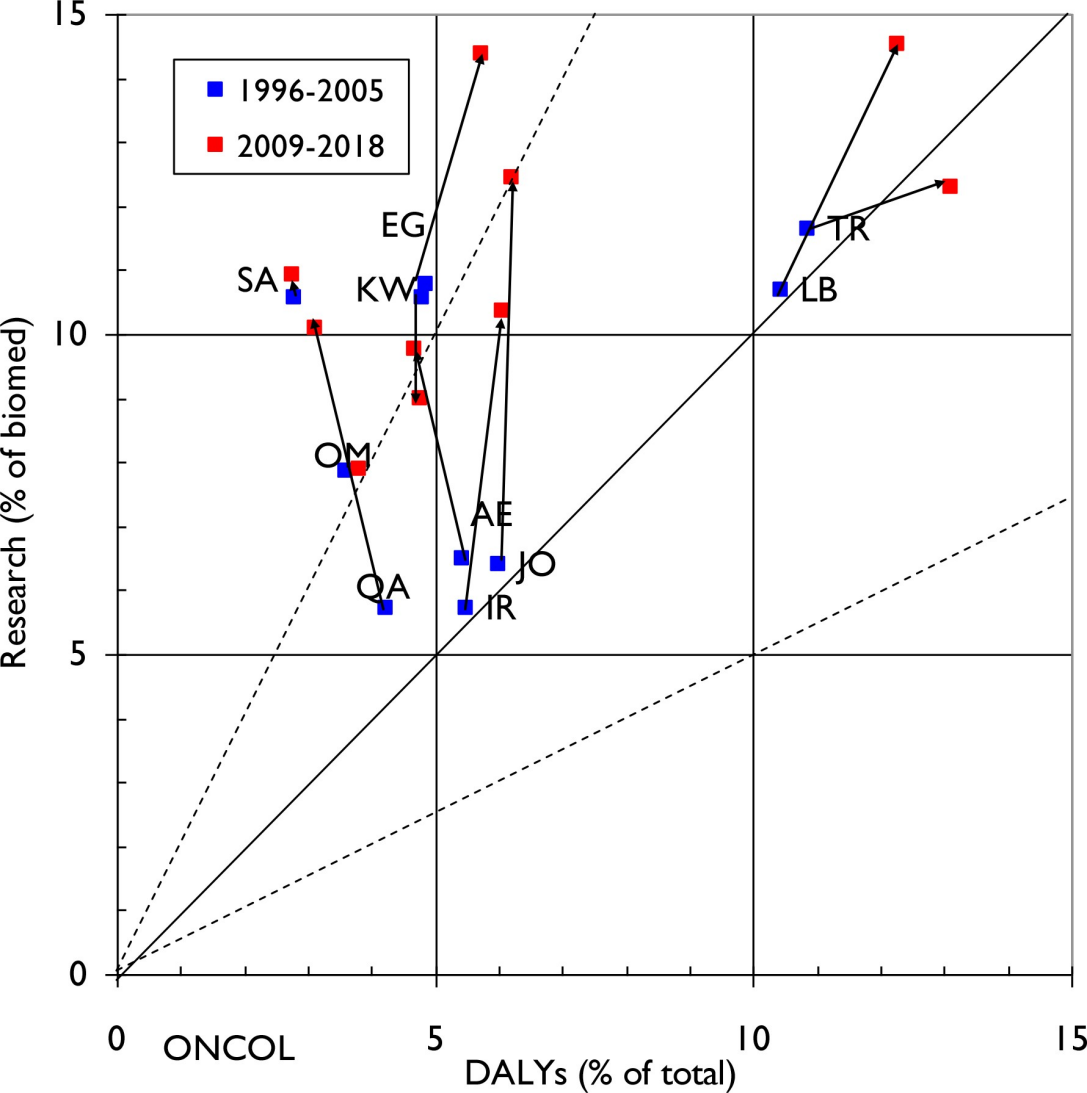
Graph demonstrating relative commitment of each ME country to cancer research (proportion of papers in oncology out of total for biomedicine) compared to its diabetes specific disease burden (measured as a percentage of DALYS attributable to oncology relative to total country-specific DALYS) for two 10-year time periods (1996–2005 and 2009–18). Arrows demonstrate change in direction of commitment to particular NCD domain research in the two time periods. The solid diagonal line represents exact equivalence of percentages, and the dashed lines represent values of research that are just twice, or half, the amounts corresponding to the percentage of DALYs.

**Fig 4:**
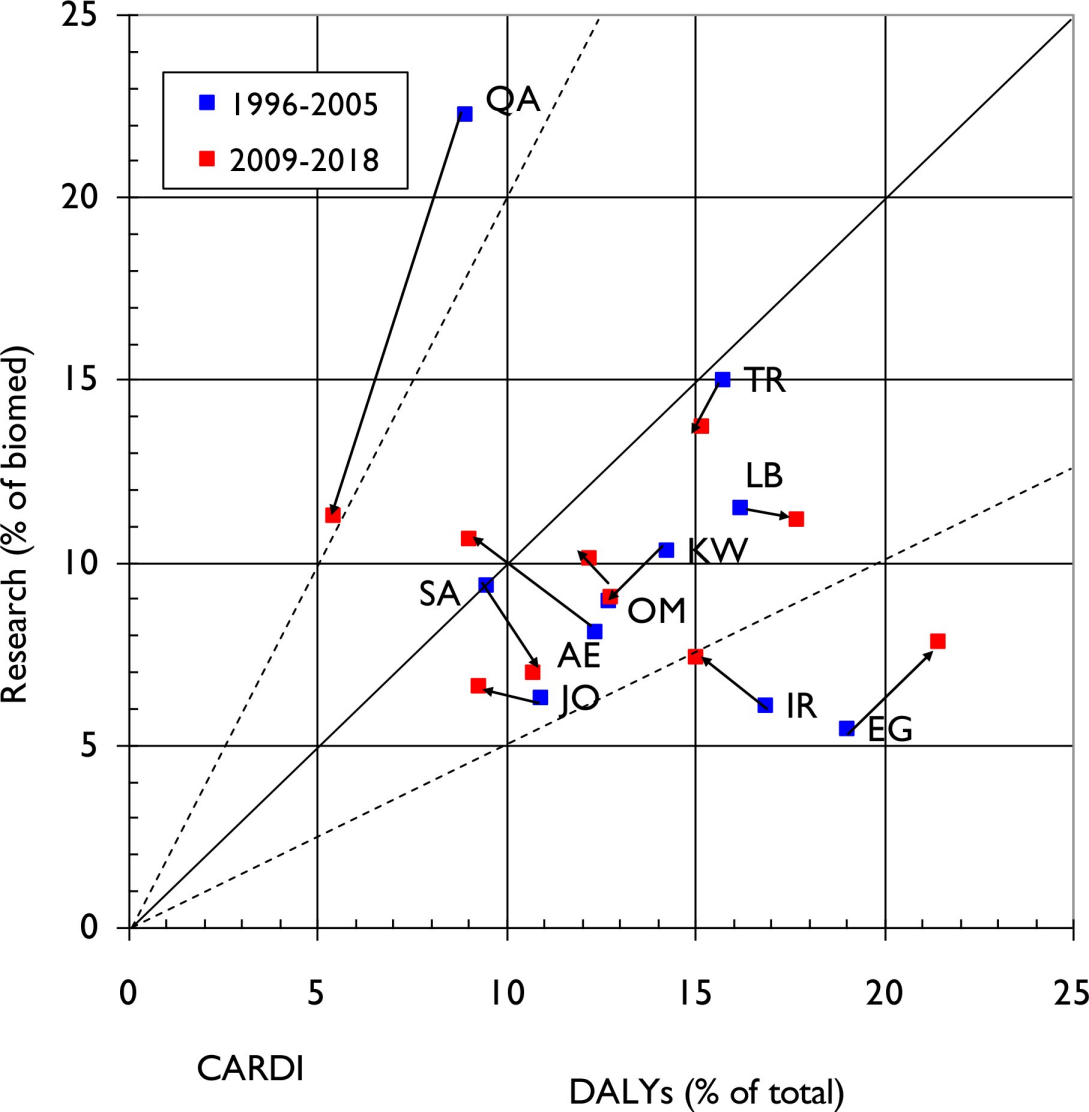
Graph demonstrating relative commitment of each ME country to cardiology research (proportion of papers in cardiology out of total for biomedicine) compared to its diabetes specific disease burden (measured as a percentage of DALYS attributable to cardiology relative to total country-specific DALYS) for two 10-year time periods (1996–2005 and 2009–18). Arrows demonstrate change in direction of commitment to particular NCD domain research in the two time periods. The solid diagonal line represents exact equivalence of percentages, and the dashed lines represent values of research that are just twice, or half, the amounts corresponding to the percentage of DALYs.

**Fig 5:**
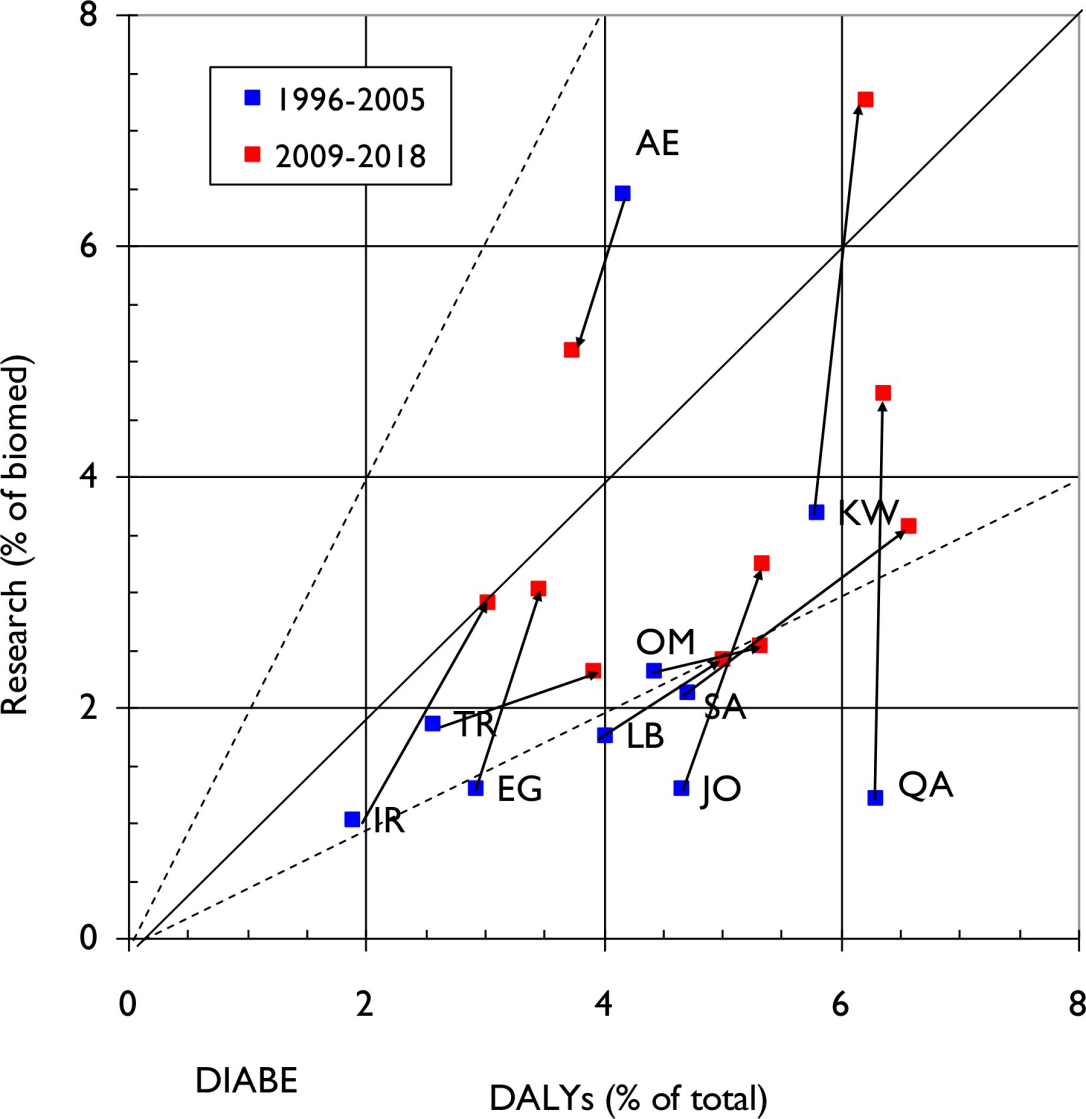
Graph demonstrating relative commitment of each ME country to diabetes research (proportion of papers in diabetes out of total for biomedicine) compared to its diabetes specific disease burden (measured as a percentage of DALYS attributable to Diabetes relative to total country-specific DALYS) for two 10-year time periods (1996–2005 and 2009–18). Arrows demonstrate change in direction of commitment to particular NCD domain research in the two time periods. The solid diagonal line represents exact equivalence of percentages, and the dashed lines represent values of research that are just twice, or half, the amounts corresponding to the percentage of DALYs.

This dynamic is illustrated in **Figs 3, 4 and 5** for the three NCDs. The arrows from blue squares (1996–2005) to red squares (2009–18) show the changes in both disease burden (percentage of the total DALYs) and in research output (percentage of biomedical research). One could expect that the country-level research outputs within each NCD would be approximately proportional to the relevant disease burden, in which case the spots should all lie on or close to the diagonal.

**Fig 3** covers cancer research and shows that it is given significant research prioritisation relative to its disease burden in all the ten MENA countries except for Turkey (TR) in the last decade (spots lying above the line). For several countries the commitment to cancer research as measured by research outputs is becoming greater: Jordan (JO), the United Arab Emirates (AE), Qatar (QA), Iran (IR) and Lebanon (LB) all had their output approximately in balance with the burden in 1996–2005, but they did a lot more cancer research in 2009–18 despite the burden being little changed.

**Fig 4** covers cardiovascular research and shows a more mixed picture. It appears to have relatively low research prioritisation (spots below the diagonal) relative to its health burden in all ten MENA countries except for Qatar (QA), where it clearly was highly prioritised with 22% of total biomedical research output in 1996–2005. However, for most of the countries, the red spots are closer to the diagonal line than the blue spots, showing that both the amount of research has increased across the two decades (apart from in Saudi Arabia (SA), Kuwait (KW) or Turkey (TR)) and that the relative cardiovascular disease burden is going down except in Egypt (EG), Lebanon (LB) and Saudi Arabia (SA).

**Fig 5** covers diabetes and shows that the burden of disease is increasing in all ten MENA countries apart from the United Arab Emirates (AE) for which diabetes research has been a major research priority when compared with the other nine countries during the time period of analysis. The increase in diabetes-related disease burden is particularly marked in Saudi Arabia (SA), Iran (IR), Oman (OM) and Turkey (TR). With respect to research outputs, Egypt (EG), Iran (IR) Jordan (JO), and Qatar (QA) all had relatively low commitments to diabetes research in the 1996–2005 period. However, they have increased their outputs considerably in the recent decade, as has Kuwait (KW), reflecting its persistently high diabetes burden.

### NCD research levels

NCD research activity is mainly clinically oriented (RL values mostly between 1.0 and 2.0). Only Egypt appeared to do rather more basic research than the other MENA countries in the three NCDs. Whilst cardiovascular and diabetes research outputs have become slightly more clinical on average (see mean scores), cancer-related output has become more basic compared to the previous decade, as exemplified by the United Arab Emirates. There has been a noticeable shift towards basic research for oncology in Qatar. Over the two decades, research outputs in all three NCD domains have become increasingly basic in Saudi Arabia.

### Citation Impact of MENA biomedical research outputs

Between 2001 and 2010 the mean five-year citation scores for biomedical research have been steadily increasing in all ten MENA countries. There have been marked rises in citation scores in Qatar (QA), Lebanon (LB), United Arab Emirates (AE) and Oman (OM). Turkey (TR), which has the largest biomedical research output in the Middle East (Fig 1) has only achieved a marginal increase in its citation scores over this time period, and has the lowest citation scores overall.

### International collaboration for the three NCD domains

**Fig 6:**
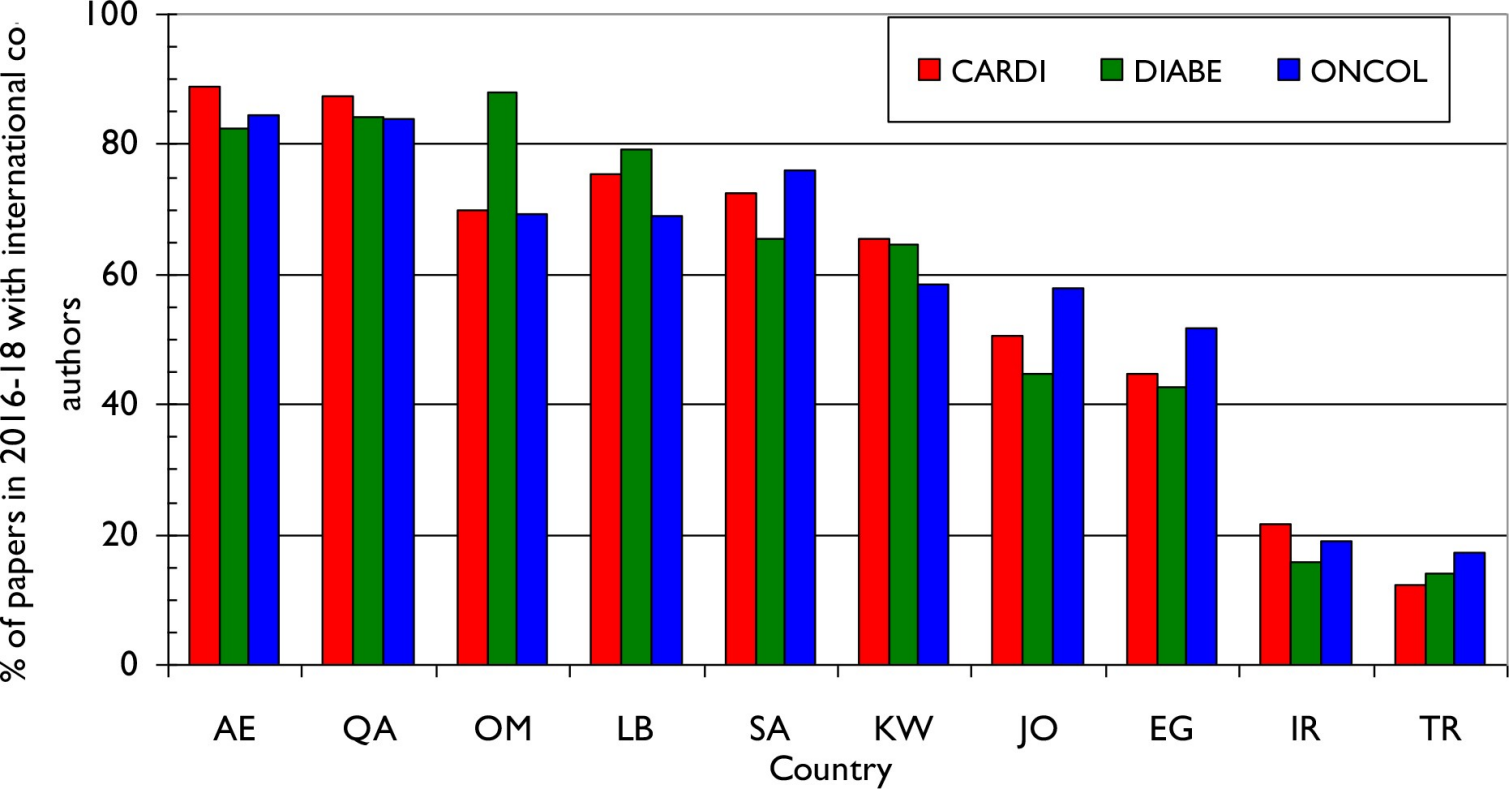
Chart showing the percentage of research output for each IW country in the three NCD domains for 2016–18 that had at least one international co-author.

We obtained the numbers of internationally co-authored research papers in the three subject areas from each of the ten MENA countries between 2016–18. There is substantial variation in the percentage of foreign involvement **(Fig 6**) with greater proportional collaboration seen in those countries that have smaller output as exemplified by Qatar (QA), United Arab Emirates (AE), Oman (OM) and Lebanon (LB). Conversely, it appears that those MENA countries with larger outputs such as Iran (IR) and Turkey (TR) collaborate less internationally. This is particularly marked for Turkey (TR), with an average of only 15% foreign contribution across all three domains of NCD research.

**Fig 7:**
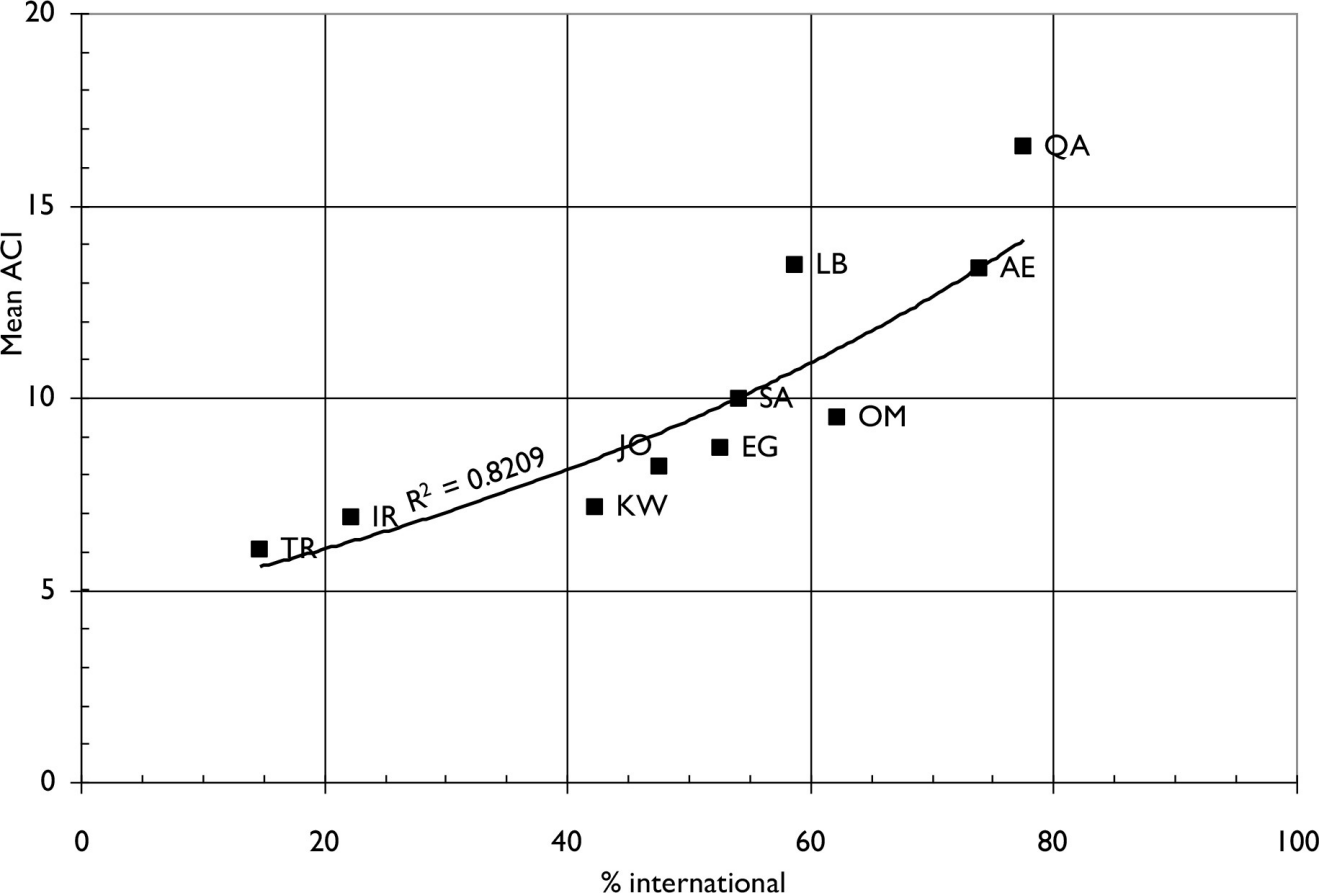
The relationship between mean five-year citation counts (ACI) for 2009–10 biomedical research papers and the percentages of these papers that are international for the ten IW countries.

**Fig 7** shows the relationship between international research collaboration in 2009–10 for the ten MENA countries and the influence of their published biomedical research, as measured by five-year citation counts in the WoS. The citation counts appear to be well correlated with the extent of international collaboration, with those countries that have a larger proportion of their papers co-authored with other countries having higher mean citation counts. Both Qatar (QA) and Lebanon (LB) have higher citation scores than expected on the basis of the regression line.

We examined the amount of collaboration with countries outside the ten MENA states between 2011–18. The Salton Index values (see methods section) for the 10 most biomedically research-active countries are shown in **Table 2**. The United States and United Kingdom are the dominant research partners across the region followed by Germany. France is notable for its collaboration with Lebanon, and the United Kingdom for its collaboration with Qatar. Egypt collaborates extensively with the United States and Germany. Saudia Arabia, appears to collaborate with a number of major research active countries including, the US, UK, Canada, Australia and India. Italy is noticeable for its collaboration with Turkey, which collaborates little with other international partners.

**Table 2:** Salton Indexes for each MENA country's biomedical research papers from 2011–18 that are co-authored with one of 10 other leading countries.

	*AE*	*EG*	*IR*	*JO*	*KW*	*LB*	*OM*	*QA*	*SA*	*TR*
*US*	1.68	2.19	1.69	1.27	0.81	1.93	0.63	1.96	2.91	1.93
*CN*	0.55	0.93	0.56	0.27	0.23	0.39	0.45	0.74	1.52	0.60
*UK*	1.78	1.94	1.46	1.16	0.93	1.17	0.97	2.71	2.74	1.74
*DE*	1.10	2.32	1.21	0.86	0.50	0.79	0.83	1.45	1.91	1.94
*JP*	0.50	1.64	0.42	0.44	0.31	0.55	0.30	0.37	1.05	0.56
*CA*	1.36	1.31	1.49	0.87	0.86	1.36	0.69	1.32	2.54	0.84
*IT*	1.00	1.45	1.34	0.70	0.49	1.38	0.61	1.66	1.73	2.15
*FR*	1.00	1.30	0.96	0.46	0.44	3.47	0.61	1.84	1.57	1.73
*AU*	1.30	0.76	1.51	0.88	0.81	0.77	0.71	1.73	2.01	0.80
*IN*	1.47	1.35	0.90	0.56	0.43	0.59	1.61	1.20	4.56	0.88

US = United States, CN = China, UK = United Kingdom, DE = Germany, JP = Japan, CA = Canada, IT = Italy, FR = France, AU = Australia, ES = Spain, IN = India. Cells with values > 2 tinted pale green; cells with values < 0.5 tinted pale yellow.

We investigated which MENA countries the ten MENA countries collaborated with in the eight years, 2011–18. Much of their international collaboration was with each other, and **Table 3** shows the Salton Index for each of the 10 MENA countries with a co-author from one of the other nine. Both Saudi Arabia (SA) and Egypt (EG) appear to be the dominant research collaborators across the MENA region. As well as contributing to each other’s research output they are also key collaborators for Oman (OM), Qatar (QA), and Jordan (JO). Turkey (TR) and Iran (IR), which are the two leading research active countries in the area, show little evidence of collaboration.

**Table 3:** Salton Indexes for each MENA country's international biomedical research papers from 2011–18 that are co-authored with one of the other nine. Cells with values > 2 tinted pale green; cells with values > 5 tinted bright green.

	*AE*	*EG*	*IR*	*JO*	*KW*	*LB*	*OM*	*QA*	*SA*	*TR*
*AE*		3.2	0.8	4.4	3.8	3.7	6.3	4.6	4.1	0.8
*EG*			1.2	1.9	3.1	2.3	2.3	3.5	20.3	1.4
*IR*				0.6	0.6	0.7	0.9	1.5	1.0	1.2
*JO*					1.6	2.8	2.0	2.4	5.0	0.6
*KW*						1.9	3.1	3.2	3.0	0.5
*LB*							1.7	3.1	2.4	0.7
*OM*								4.0	3.7	0.6
*QA*									3.1	1.3
*SA*										1.5

## Discussion

The majority of the MENA countries have shown a rapid improvement in their health status over the last three decades, primarily through a reduction in mortality from communicable diseases. However, their approach to managing the ever-increasing burden of NCDs has largely focused on a curative model with a proposed expansion of health care resources and uptake of new technologies, rather than prevention and health promotion services [25]. It is imperative that there is capacity building to strengthen biomedical research in the MENA region to address the rising tide of NCDs by improving international collaboration, supporting investment in research and training, and improving the research environment [26].

The increase in research activity in NCDs across the MENA region during the time period of analysis may signal both an increasing focus on NCDs which reflects general global trends, and greater investment in research in some countries. For example, in Qatar there has been a substantial increase in research funding, $100 million over 10 years, which perhaps explains its recent rapid improvement in research outputs and citation impact [27]. It has also sought to align its clinical and academic health development strategies through the creation of academic health centers and partnerships [28].

However, our analysis shows that some MENA countries, particularly Oman, Qatar, Kuwait and the United Arab Emirates are still substantially under-investing in biomedical research relative to their GDP. There is also continued evidence of low political commitment in the region, poor financial support for researchers, as well as poor access to funding for NCD research despite some major potential sources [25].

This is illustrated by the fact that 4.6% of the world’s financial resources are produced in the Eastern Mediterranean region member states, yet the share of health research resources in the region is only 0.6% (one-seventh of the region’s financial resources)[29]. The recent UNESCO report estimated that the ratio of gross domestic expenditure on R&D (GERD) to GDP was very low in the region, and has been on average 0.3%, 97% of which was provided by public resources. The positive point observed in this report is the increase in R&D investment from a few countries. For example, this ratio was 0.03% in Tunisia in 1996, but had reached 1% by 2000. In countries such as Iran and Egypt, there is a 5-year plan to reach 3% and 1% respectively by 2012. Among the wealthier nations, Qatar has had a plan to reach 2.8% in 2011 [29].

### NCD priorities in the region

In terms of investment on particular NCDs, we note the relatively greater investment on cancer research compared with diabetes or cardiovascular disease in most MENA countries, despite cardiovascular disease causing the greatest health-related burden (GBD, 2013). In addition, the Gulf Cooperative States (Oman, UAE, Qatar) countries face a continued rise in diabetes incidence, yet our findings show that, compared to their wealth, there is relatively less research prioritisation on addressing the burden.

The research outputs across these three main NCDs are becoming increasingly clinically focused across the last two decades. This is in contrast to findings across Europe which demonstrates that some NCD outputs are moving in the opposite direction towards more basic science research, perhaps as a result of the rising costs of clinical trials [15].

### International collaboration

One mechanism for raising the profile and citation impact of research is through international collaboration. MENA countries vary substantially in the level of intra-regional and international collaboration. Our findings illustrate that internationally-co-authored publications within the region included researchers predominantly from the USA, followed by the UK, Germany and France. This is in keeping with other studies which have shown that approximately 45% of all multinational publications include a US author [22].

We also demonstrate a strongly positive association between international collaboration and higher impact publications (as measured by citation counts) in the MENA region, irrespective of the volume of research output by individual countries. This association has been demonstrated previously, and supports the role of international collaboration in developing a country’s research influence and infrastructure [22].

While international collaboration may be seen as a research strength, these associations may mask more complex underlying mechanisms. For example, in countries, such as Iran and Turkey, which collaborate significantly less than other countries in the region but are amongst the largest producers of biomedical research, there is greater investment in research and thus a more developed infrastructure. As a result, researchers in these countries may choose to publish their research in regional journals with a low impact internationally in order to more effectively reach local policy-makers and effect change at a local level. This mechanism may also explain in part the low citation impact in some countries with the highest number of outputs, but it may also reflect an international bias in favor of Western research.

There are other caveats with this type of analysis. In particular, that the notion of collaboration does not guarantee that this is a partnership of equals or will necessarily result in building research capacity and capability. This is more likely to be the case if partnership involves merely the provision of “local” data or tissue samples for research to be done. In addition, as well as co-authorship, collaboration may be achieved through sponsorship and funding, which we have not specifically analyzed.

### Investment in training

To build a sustainable and effective research infrastructure, and tackle NCDs across the MENA region there is a pressing need to increase the cadre of national researchers, and health workers [21]. On average, there are only 25 health workers per 100,000 people in countries within the Organisation for Islamic Cooperation (OIC) (a political bloc that includes the MENA region). This is only slightly higher than the critical threshold of 23 per 100,000 needed to deliver basic health services and compares with 41 per 100,000 in non-OIC countries (OIC health report 2015). Further information on the number of academic researchers would also help to inform policy but this is currently not available.

Research seeking to understand the barriers to building research capacity, particular around creating a skilled workforce remains limited and further investigation is required across the MENA region. A possible factor is that research training is generally not a focus of the education that health workers receive and the reality is that they are often overloaded with routine clinical work, without either the basic skills or time for research [30].

On a more general level, these countries lack the depth and breadth of higher education systems to generate a sustainable knowledge economy. In part, this is due to their relatively recent entry into the knowledge economy; however, many countries in the region have started to invest in their education infrastructure [31]. In addition, there is increasing awareness especially amongst national governments that there is too much dependence on international staff, especially in the fields of science and technology. In order to nationalize the research labour force, there has to be structural reform with the introduction of clearly defined policies that focus on education, training, transfer of knowledge into ‘home grown’ NCD research [32].

### Limitations of this study

The present study has both strengths and limitations. The analysis has been undertaken on an individual country basis and findings with respect to country outputs are potentially skewed depending on the size of the population and resources of individual countries. In addition, MENA countries which conduct many collaborative multinational studies may appear to produce comparatively fewer research outputs on the basis of fractional counts, compared to Iran and Turkey, for instance. We have used citation frequency as a proxy indicator for quality of research and dissemination of scientific findings. However, a true evaluation of the scientific quality of publications cannot be achieved without an independent and dedicated assessment of their merit. Furthermore, citation frequency cannot determine whether a publication changes practice and improves population health[33]. We also acknowledge that the proportion of published studies that are trials is another mechanism for understanding the RnD infrastructure of each country, however this was not included within our analysis.

We have selected publications available in the WoS for analysis, and it is therefore likely that some research output in national language journals has not been included, which could affect our results. In addition, as with any bibliometric evaluation, it is not possible to guarantee inclusion of all relevant papers. However, we have allowed for the lack of precision and recall of the three filters by making corrections to the numbers of papers.

Finally, although we examined the outputs of 10 MENA countries, our analysis may not hold for the other countries within the region. We selected “bellweather” countries with the highest volume of research outputs during the time-period of analysis. The additional micro-analysis of lower research output countries would not inform the wider discussion regarding workforce, training and investment in the region. For example, after excluding Iraq, Syria and Yemen due to their political instability/current ongoing conflict–the next highest research producers (according to volume of outputs) are Bahrain, Libya, Azerbaijan, Palestine, Afghanistan whichbetween 2009–2018 produced 4343 publications (approximately 1.1% of the total whenconsidering the overall total including the 10 main research active countriesn = 396,228). We also considered the research ecosystem to enable comparison and therefore did not include Tunisia, Morocco and Algeria which are geographically distinct north African countries who’s regional research connections are distinct from the rest of the MENA region.

## Conclusion

The increase in research activity in NCDs across the MENA region during the time period of analysis may signal both an increasing focus on NCDs which reflects general global trends, and greater investment in research in some countries. The analysis demonstrates heterogeneity in research outputs relative to country wealth, population size as well as variation in prioritization of research into cancer, cardiovascular disease and diabetes across MENA countries, with no clear correlation with disease burden. MENA countries in this evaluation vary greatly in the extent of their collaboration with other countries in the region and that of international partners, particularly the US and UK.

A number of MENA countries have exceptional levels of wealth which could, with the right policy measures and public health strategies, offer an unprecedented opportunity to build vital NCD research systems [34]. This research is expected to support research capacity building in the region, by highlighting countries that still do not sufficiently invest in RnD relative to their GDP, by focusing research prioritization towards high burden NCDs in the region, and by demonstrating how research impact can be improved through international collaborations.

## Supporting information

S1 Fig Outputs of ONCOL (cancer research) papers (integer counts) from 10 IW countries, 1991–2018, presented as three-year running means on a logarithmic scale (Y axis). (DOCX)

S2 Fig Outputs of CARDI (cardiovascular disease research) papers (integer counts) from 10 IW countries, 1991–2018, presented as three-year running means on a logarithmic scale (Y axis). (DOCX)

S3 Fig Outputs of DIABE (diabetes research) papers (integer counts) from 10 IW countries, 1991–2018, presented as three-year running means on a logarithmic scale (Y axis). (DOCX)

S1 File (PDF)
