# Supplementary material for: The Profile of Non-Communicable Disease (NCD) research in the Middle East and North Africa (MENA) region: Analyzing the NCD burden, research outputs and international research collaboration
Source: PLoS One. 2020 Apr 27;15(4):e0232077. doi: 10.1371/journal.pone.0232077 (PMC7185716; doi:10.1371/journal.pone.0232077)
Supplement: S2 Fig — (DOCX) [file pone.0232077.s002.docx]

**Supplementary Figure 2**. Outputs of CARDI (cardiovascular disease research) papers (integer counts) from 10 IW countries, 1991-2018, presented as three-year running means on a logarithmic scale (Y axis).
