# Supplementary material for: The Profile of Non-Communicable Disease (NCD) research in the Middle East and North Africa (MENA) region: Analyzing the NCD burden, research outputs and international research collaboration
Source: PLoS One. 2020 Apr 27;15(4):e0232077. doi: 10.1371/journal.pone.0232077 (PMC7185716; doi:10.1371/journal.pone.0232077)
Supplement: S1 File — (PDF) [file pone.0232077.s004.pdf]

Filter for BIOMED

AD=(ABBOTT or ABBVIE or ACTELION or AGEING or AGING or AIDS or ALEXION or ALLERG* or AMGEN or AN*ESTHE* or ANAT or ANIM or ASTELLAS or ASTRAZENECA or AVENTIS or BAYER or BETHESDA or BIOCH*M or BIOENGN or BIOGEN or BIOINFORMAT or BIOMARIN or BIOMED* or BIOPHYS or BIOQUIM or BIOSCI or BIOSTAT or BIOTEC* or BOEHRINGER or BRAIN or BRISTOL-MYERS or BMS or CANC or CARDIAC* or CARDIO* or CARE or CEL*ULA*R* or CELGENE or CELL or CHEST or CHILD* or CHU-* or CILAG or CLIN or COGNIT or CRIT or CSL or CTR-DIS-CONTROL* or CYTO* or DAIICHI or DENT or DERMATOL* or DIABET* or DIAGNOST or DIS or DISORDER* or DRUG* or EISAI or ELI-LILLY or EMERGENCY or ENDOCRIN* or EPIDEM* or EYE or FAMILY or FARMAC* or GASTRO* or GENE or GENENTECH or GENET or GENOM* or GENZYME or GERIATR or GILEAD or GLAXO* or GYN*ECOL or H*EMATO* or HEAD or HEART or HEPAT* or HISTO* or HLTH* or HOP or HOP-* or HOSP* or HUMAN or HYG or HYPERTENS or IMMUN* or INFECT or INSERM* or IRCCS* or JANSSEN or JOHNSON-&-JOHNSON or KAROLINSKA* or KLIN* or LILLY or LIVER or MACROMOL or MAXILLOFACIAL or MED or MED-* or MEM-* or MERCK or METAB or MICROBI*L or MRC or MSD or MYLAN or NCI or NECK or NEONAT*L or NEPHROL* or NEURO* or NIH or NOVARTIS or NOVO-NORDISK or NURSING or NUTR or OBSTET or OCCUPAT or ONCOL* or OPHTHALMOL or ORAL or ORGANON or ORTHO or ORTHOP*ED or OSPED-* or OTOLARYNGOL or OTORHINOLARYNGOL or OTSUKA or P*EDIAT or PARASITOL or PATHO* or PFIZER or PHARM* or PHYSIO* or POLI*LIN or POPULAT or PREVENT or PROT or PSYCH* or PULM* or RADIODIAG or RADIOL or REGENERON or REHABIL or RENAL or REPROD or RESP or RH*UMATOL or ROCHE or SANOFI or SCHERING-PLOUGH or SERVIER or SHIRE or SURG or TAKEDA or TEVA or THERAP* or THORAC or TOXI*OL or TRANSPLANT* or TUMOR* or UCB or UROL or VASC or VERTEX-PHARM* or VET or VET-* or VIROL or WOMEN* or WYETH)

Filter for CARDI

(SO=(ACTA-CARDIOLOGICA* or ADVANCES-IN-CARDIOLOGY or AMERICAN-HEART-JOURNAL or AMERICAN-JOURNAL-OF-HYPERTENSION or AMERICAN-JOURNAL-OF-PHYSIOLOGY-HEART-AND-CIRCULATORY-PHYSIOLOGY or ANGIOLOGY or ANNALES-DE-CARDIOLOGIE-ET-D'ANGEIOLOGIE or ARCHIVES-DES-MALADIES-DU-COEUR-ET-DES-VAISSEAUX or ARCHIVES-OF-CARDIOVASCULAR-DISEASES or ARTERIOSCLEROSIS-THROMBOSIS-AND-VASCULAR-BIOLOGY or ATHEROSCLEROSIS* or BMC-CARDIOVASCULAR-DISORDERS or CARDIOLOGY* or CARDIORENAL-SYNDROMES-IN-CRITICAL-CARE or (CARDIOVASCULAR* NOT DIABETOLOGY) or CATHETERIZATION-AND-CARDIOVASCULAR-INTERVENTIONS or CEREBROVASCULAR-DISEASES or CLINICAL-CARDIOLOGY or CORONARY-ARTERY-DISEASE or CURRENT-ATHEROSCLEROSIS-REPORTS or CURRENT-HYPERTENSION-REPORTS or CURRENT-TOPICS-IN-DEVELOPMENTAL-BIOLOGY or ECHOCARDIOGRAPHY-A-JOURNAL-OF-CARDIOVASCULAR-ULTRASOUND-AND-ALLIED-TECHNIQUES or EUROPACE or EUROPEAN-HEART-JOURNAL* or EUROPEAN-JOURNAL-OF-CARDIO-THORACIC-SURGERY or EUROPEAN-JOURNAL-OF-VASCULAR-AND-ENDOVASCULAR-SURGERY or HEART* or HERZ or HYPERTENSION* or INTERNATIONAL-HEART-JOURNAL or INTERNATIONAL-JOURNAL-OF-CARDIOLOGY or JACC-CARDIOVASCULAR* or JAPANESE-CIRCULATION-JOURNAL-ENGLISH-EDITION or JOURNAL-DES-MALADIES-VASCULAIRES or JOURNAL-OF-CARDIAC-SURGERY or JOURNAL-OF-CARDIOLOGY

or JOURNAL-OF-CARDIOPULMONARY-REHABILITATION-AND-PREVENTION or JOURNAL-OF-CARDIOTHORACIC* or JOURNAL-OF-CARDIOVASCULAR* or JOURNAL-OF-CLINICAL-LIPIDOLOGY or JOURNAL-OF-ENDOVASCULAR-THERAPY or JOURNAL-OF-HEART* or JOURNAL-OF-HUMAN-HYPERTENSION or JOURNAL-OF-HYPERTENSION or JOURNAL-OF-INVASIVE-CARDIOLOGY or JOURNAL-OF-MOLECULAR-AND-CELLULAR-CARDIOLOGY or JOURNAL-OF-THE-AMERICAN-COLLEGE-OF-CARDIOLOGY or JOURNAL-OF-THE-AMERICAN-SOCIETY-OF-ECHOCARDIOGRAPHY or JOURNAL-OF-THORACIC-AND-CARDIOVASCULAR-SURGERY or JOURNAL-OF-VASCULAR-SURGERY or KARDIOLOGIYA or MICROVASCULAR-RESEARCH or NETHERLANDS-HEART-JOURNAL or PACE-PACING-AND-CLINICAL-ELECTROPHYSIOLOGY or PEDIATRIC-CARDIOLOGY or PROGRESS-IN-CARDIOVASCULAR-DISEASES or REVIEWS-IN-CARDIOVASCULAR-MEDICINE or REVISTA-ESPANOLA-DE-CARDIOLOGIA or SCANDINAVIAN-CARDIOVASCULAR-JOURNAL or SEMINARS-IN-THROMBOSIS-AND-HEMOSTASIS or STROKE or TEXAS-HEART-INSTITUTE-JOURNAL or THORACIC-AND-CARDIOVASCULAR-SURGEON or THROMBOSIS* or TRENDS-IN-CARDIOVASCULAR-MEDICINE or VASCULAR* or ZEITSCHRIFT-FUR-KARDIOLOGIE) or TI=(ACS or (AMBULATORY AND BLOOD) or ANGINA or ANEURYSM* or ANGIOGENIC or ANGIOGRAPH* or ANGIOPLAST* or ANGIOTENSIN or ANKLE-BRACHIAL* or ANTIHYPERTENS* or ANTIARRHYTHM* or AORT* or (APICAL AND BALLOONING) or (ARTERY AND BYPASS) or ARRHYTHM* or ARTERIOLAR or ATHEROSCLERO* or ATORVASTATIN or ATRIAL or ATRIOVENTRICULAR or (BARE* AND METAL AND STENT*) or (BETA AND BLOCK*) or (BLOOD AND PRESSURE) or BNP or BRUGADA or ((BYPASS AND SURGERY) NOT GASTRIC) or CABG or CARDIAC or CARDIO* or CAROTID or CEREBROVASCULAR or CHD or CLOPIDOGREL or CORONARY or DABIGATRAN or DIASTOLIC or DEFIBRILLAT* or DIGITALIS or DIGOXIN or (DRUG* AND ELUTI* AND STENT*) or (DUCTUS AND ARTERIOS*) or EBSTEIN or ECG or ECHOCARDIOGR* or EDRF or EKG or ELECTROCARDIO* or EMBOLISM or ENDOCARDI* or ENDOMYOCARD* or EPICARDI* or (FACTOR AND (VIII or IX)) or FIBRILLATION or HEMANGIOMA* or HEMODYNAMICS or HEART or HEARTBEAT or HEARTMATE or (HEARTS NOT MINDS) or HEPARIN or HYPERCHOLESTEROL* or (HYPERTENS* NOT PORTAL) or HYPERLIPID* or ICD or INFARCT* or INTERATRIAL or INTERVENTRICULAR or INTRACARDIA* or INTRACORONARY or INTRAVASCULAR or INOTROPIC or ISCHEMIA or ISCHEMIC or KAWASAKI* or LOVASTATIN or MARFAN* or MITRAL or MICROCIRCULAT* or MICROVASCULA* or MYOCARDI* or NITROGLYCERIN or NSTEMI or PAPAVERINE or PACEMAKER* or PCI or PERICARDI* or PRASUGREL or (PULMONARY AND VALVE) or (PULMONARY AND EMBOLISM) or QT or QTC or REPERFUSION or REVASCULARI* or ROSUVASTATIN or SAPHENOUS or (ST AND SEGMENT) or SIMVASTATIN or SINOATRI* or STATIN or STEMI or (STROKE NOT (2-STROKE or 4-STROKE or ENGINE or MOTOR)) or SYSTOLIC or TACHYCARDI* or TAKAYASU* or TAKOTSUBO or TETRALOGY*FALLOT or THROMBOS* or THROMBEMBOL* or THROMBOLY* or THROMBOXANE or TICAGRELOR or TRICUSPID or TRIGLYCERIDE* or TROPONIN or (VASCULAR* NOT (ALZHEIMER* or CANCER or CARCINOMA or DIABET* or RETINA* or TUMOR)) or VALVUL* or VENTRIC* or (VEIN* NOT (MINERAL or PORTAL or UMBILICAL)) or (VENA AND CAVA) or VASOCONSTRICT* or VASODILAT* or VASOMOTOR or VASOACTIVE or VENTRICULAR or WOLFF-PARKINSON-WHITE) or (TI=PLATELET* NOT SO=BLOOD)) not (TI=(ALZHEIMER* or ANTIMALARIA* or DEMENTIA or FALCIPARUM or MALARIA* or OPTIC* or RETINA*))

Filter for DIABE

(SO=(ACTA-DIABETOLOGICA* or CARDIOVASCULAR-DIABETOLOGY or CURRENT-DIABETES-REPORTS or DIABETE-METABOLISME or DIABETES or DIABETES-METABOLISM or DIABETES-VASCULAR-DISEASE-RESEARCH or DIABETES-AND-METABOLISM or DIABETES-AND-THE-KIDNEY or DIABETES-CARE or DIABETES-EDUCATOR or DIABETES-MELLITUS* or DIABETES-METABOLISM or DIABETES-METABOLISM-RE* or DIABETES-NUTRITION* or DIABETES-OBESITY-METABOLISM or DIABETES-RESEARCH-AND-CLINICAL-PRACTICE or DIABETES-REVIEWS or DIABETES-STOFFWECHSEL-UND-HERZ or DIABETES-TECHNOLOGY-THERAPEUTICS or DIABETES-VASCULAR-DISEASE-RESEARCH or DIABETIC-MEDICINE or DIABETOLOGE or DIABETOLOGIA or DIABETOLOGIE-UND-STOFFWECHSEL or DIABETOLOGY-METABOLIC-SYNDROME or EXPERIMENTAL-AND-CLINICAL-ENDOCRINOLOGY-DIABETES or EXPERIMENTAL-AND-CLINICAL-ENDOCRINOLOGY-AND-DIABETES or EXPERIMENTAL-DIABETES-RESEARCH or IMMUNOLOGY-OF-DIABETES* or INTERNATIONAL-JOURNAL-OF-DIABETES-IN-DEVELOPING-COUNTRIES or JOURNAL-OF-DIABETES-AND-ITS-COMPLICATIONS or JOURNAL-OF-DIABETES-INVESTIGATION or METABOLISMES-HORMONES-DIABETES-ET-NUTRITION or PEDIATRIC-DIABETES or PRIMARY-CARE-DIABETES) or TI=(ACARBOSE or ANTIDIABETIC* or DCCT or DIABET* or GLP or GLUT-1 or GLUT-2 or GLUT-3 or GLUT-4 or GLUT1 or GLUT2 or GLUT3 or GLUT4 or GLUCOSE-MONITOR* or GLUCOSE-TRANSPORTER-1 or GLUCOSE-TRANSPORTER-2 or GLUCOSE-TRANSPORTER-3 or GLUCOSE-TRANSPORTER-4 or IDDM or INSULIN-RESIST* or MICRAL or MODY or MODY1 or MODY2 or MODY3 or MODY4 or MODY5 or NESIDIOBLAST* or NIDDM or NOD-MICE or NOD-MOUSE or PHOGRIN or PREDIABETIC or PROINSULIN or THIAZOLIDINEDIONE*)) not (TI=((CANCER or CARCINOMA) NOT DIABET*))

Filter for ONCOL

(SO=(ACTA-ONCOLOGICA or ADVANCES-IN-CANCER-BIOMARKERS-FROM-BIOCHEMISTRY-TO-CLINIC-FOR-A-CRITICAL-REVISION or ADVANCES-IN-CANCER-RESEARCH or ADVANCES-IN-IMMUNOLOGY or AMERICAN-JOURNAL-OF-CANCER-RESEARCH or AMERICAN-JOURNAL-OF-CLINICAL-ONCOLOGY-CANCER-CLINICAL-TRIALS or ANNALS-OF-ONCOLOGY or ANNALS-OF-SURGICAL-ONCOLOGY or ANTI-CANCER-AGENTS-IN-MEDICINAL-CHEMISTRY or ANTI-CANCER-DRUGS or ANTICANCER-RESEARCH or APPLICATIONS-OF-VIRUSES-FOR-CANCER-THERAPY or ASIAN-PACIFIC-JOURNAL-OF-CANCER-PREVENTION or ASIA-PACIFIC-JOURNAL-OF-CLINICAL-ONCOLOGY or BIOCHIMICA-ET-BIOPHYSICA-ACTA-REVIEWS-ON-CANCER or BIOLOGICAL-BASIS-OF-ALCOHOL-INDUCED-CANCER or BLOOD-CANCER-JOURNAL or BMC-CANCER or BRAIN-TUMOR-PATHOLOGY or BREAST-CANCER or BREAST-CANCER-RESEARCH or BREAST-CANCER-RESEARCH-AND-TREATMENT or BRITISH-JOURNAL-OF-CANCER or BULLETIN-DU-CANCER or CA-A-CANCER-JOURNAL-FOR-CLINICIANS or CANCER or CANCER-AND-METASTASIS-REVIEWS or CANCER-BIOLOGY-THERAPY or CANCER-BIOMARKERS or CANCER-BIOTHERAPY-AND-RADIOPHARMACEUTICALS or CANCER-CAUSES-CONTROL or CANCER-CELL or CANCER-CELL-INTERNATIONAL or CANCER-CHEMOTHERAPY-AND-PHARMACOLOGY or CANCER-CONTROL or CANCER-CYTOPATHOLOGY or CANCER-DISCOVERY or CANCER-EPIDEMIOLOGY or CANCER-EPIDEMIOLOGY-BIOMARKERS-PREVENTION or CANCER-GENE-THERAPY or CANCER-GENETICS or CANCER-GENOMICS-PROTEOMICS or CANCER-IMAGING or CANCER-IMMUNOLOGY-IMMUNOTHERAPY or CANCER-IMMUNOLOGY-RESEARCH or

CANCER-INVESTIGATION or CANCER-JOURNAL or CANCER-LETTERS or CANCER-MEDICINE or CANCER-NURSING or CANCER-PREVENTION-RESEARCH or CANCER-RADIOTHERAPIE or CANCER-RESEARCH or CANCER-RESEARCH-AND-TREATMENT or CANCER-SCIENCE or CANCER-TREATMENT-REVIEWS or CARCINOGENESIS or CELL-POLARITY-AND-CANCER or CELLULAR-ONCOLOGY or CHEMOTHERAPY or CHINESE-JOURNAL-OF-CANCER or CHINESE-JOURNAL-OF-CANCER-RESEARCH or CLINICAL-&-EXPERIMENTAL-METASTASIS or CLINICAL-BREAST-CANCER or CLINICAL-CANCER-RESEARCH or CLINICAL-COLORECTAL-CANCER or CLINICAL-GENITOURINARY-CANCER or CLINICAL-JOURNAL-OF-ONCOLOGY-NURSING or CLINICAL-LUNG-CANCER or CLINICAL-LYMPHOMA-MYELOMA-LEUKEMIA or CLINICAL-ONCOLOGY or CLINICAL-TRANSLATIONAL-ONCOLOGY or CRITICAL-REVIEWS-IN-ONCOLOGY-HEMATOLOGY or CURRENT-ADVANCES-IN-OSTEOSARCOMA or CURRENT-CANCER-DRUG-TARGETS or CURRENT-ONCOLOGY or CURRENT-ONCOLOGY-REPORTS or CURRENT-OPINION-IN-ONCOLOGY or CURRENT-PROBLEMS-IN-CANCER or CURRENT-TREATMENT-OPTIONS-IN-ONCOLOGY or ENDOCRINE-RELATED-CANCER or EUROPEAN-JOURNAL-OF-CANCER or EUROPEAN-JOURNAL-OF-CANCER-CARE or EUROPEAN-JOURNAL-OF-CANCER-PREVENTION or EUROPEAN-JOURNAL-OF-GYNAECOLOGICAL-ONCOLOGY or EUROPEAN-JOURNAL-OF-ONCOLOGY or EUROPEAN-JOURNAL-OF-ONCOLOGY-NURSING or EXPERT-REVIEW-OF-ANTICANCER-THERAPY or FAMILIAL-CANCER or FUTURE-ONCOLOGY or GASTRIC-CANCER or GENES-CHROMOSOMES-CANCER or GUIDANCE-MOLECULES-IN-CANCER-AND-TUMOR-ANGIOGENESIS or GYNECOLOGIC-ONCOLOGY or HEAD-NECK-ONCOLOGY or HEMATOLOGICAL-ONCOLOGY or HEMATOLOGY-ONCOLOGY-CLINICS-OF-NORTH-AMERICA or HEREDITARY-CANCER-IN-CLINICAL-PRACTICE or HORMONES-CANCER or IMMUNITY-TO-LISTERIA-MONOCYTOGENES or INDIAN-JOURNAL-OF-CANCER or INFECTIOUS-AGENTS-AND-CANCER or INFLAMMATION-AND-CANCER or INTEGRATIVE-CANCER-THERAPIES or INTERNATIONAL-JOURNAL-OF-CANCER or INTERNATIONAL-JOURNAL-OF-CLINICAL-ONCOLOGY or INTERNATIONAL-JOURNAL-OF-GYNECOLOGICAL-CANCER or INTERNATIONAL-JOURNAL-OF-ONCOLOGY or INTERNATIONAL-JOURNAL-OF-RADIATION-ONCOLOGY-BIOLOGY-PHYSICS or JAPANESE-JOURNAL-OF-CLINICAL-ONCOLOGY or JNCI-JOURNAL-OF-THE-NATIONAL-CANCER-INSTITUTE or JOURNAL-OF-ADOLESCENT-AND-YOUNG-ADULT-ONCOLOGY or JOURNAL-OF-BONE-ONCOLOGY or JOURNAL-OF-BREAST-CANCER or JOURNAL-OF-CANCER or JOURNAL-OF-CANCER-EDUCATION or JOURNAL-OF-CANCER-RESEARCH-AND-CLINICAL-ONCOLOGY or JOURNAL-OF-CANCER-RESEARCH-AND-THERAPEUTICS or JOURNAL-OF-CANCER-SURVIVORSHIP* or JOURNAL-OF-CHEMOTHERAPY or JOURNAL-OF-CLINICAL-ONCOLOGY or JOURNAL-OF-ENVIRONMENTAL-PATHOLOGY-TOXICOLOGY-AND-ONCOLOGY or JOURNAL-OF-EXPERIMENTAL-CLINICAL-CANCER-RESEARCH or JOURNAL-OF-GERIATRIC-ONCOLOGY or JOURNAL-OF-GYNECOLOGIC-ONCOLOGY or JOURNAL-OF-HEMATOLOGY-ONCOLOGY or JOURNAL-OF-MEDICAL-IMAGING-AND-RADIATION-ONCOLOGY or JOURNAL-OF-NEURO-ONCOLOGY or JOURNAL-OF-PEDIATRIC-HEMATOLOGY-ONCOLOGY or JOURNAL-OF-PEDIATRIC-ONCOLOGY-NURSING or JOURNAL-OF-PSYCHOSOCIAL-ONCOLOGY or JOURNAL-OF-SURGICAL-ONCOLOGY or JOURNAL-OF-THE-NATIONAL-CANCER-INSTITUTE or JOURNAL-OF-THE-NATIONAL-COMPREHENSIVE-CANCER-NETWORK or JOURNAL-OF-THORACIC-ONCOLOGY or LANCET-ONCOLOGY or LEUKEMIA or LEUKEMIA-LYMPHOMA or

LEUKEMIA-RESEARCH or LUNG-CANCER or MEDICAL-ONCOLOGY or MELANOMA-RESEARCH or MICRORNA-CANCER-FROM-MOLECULAR-BIOLOGY-TO-CLINICAL-PRACTICE or MOLECULAR-CANCER or MOLECULAR-CANCER-RESEARCH or MOLECULAR-CANCER-THERAPEUTICS or MOLECULAR-CARCINOGENESIS or MOLECULAR-ONCOLOGY or NATURE-REVIEWS-CANCER or NATURE-REVIEWS-CLINICAL-ONCOLOGY or NEOPLASIA or NEOPLASMA or NEUROENDOCRINE-TUMORS-A-MULTIDISCIPLINARY-APPROACH or NEURO-ONCOLOGY or NUTRITION-AND-CANCER-AN-INTERNATIONAL-JOURNAL or ONCOGENE or ONCOGENESIS or ONCOIMMUNOLOGY or ONCOLOGIE or ONCOLOGIST or ONCOLOGY or ONCOLOGY-LETTERS or ONCOLOGY-NEW-YORK or ONCOLOGY-NURSING-FORUM or ONCOLOGY-REPORTS or ONCOLOGY-RESEARCH or ONCOLOGY-RESEARCH-AND-TREATMENT or ONCOTARGET or ONCOTARGETS-AND-THERAPY or ONKOLOGE or ONKOLOGIE or ORAL-ONCOLOGY or PATHOLOGY-ONCOLOGY-RESEARCH or PEDIATRIC-BLOOD-CANCER or PEDIATRIC-HEMATOLOGY-AND-ONCOLOGY or PIGMENT-CELL-MELANOMA-RESEARCH or PROGRESS-IN-TUMOR-RESEARCH or PROSTATE-CANCER-AND-PROSTATIC-DISEASES or PSYCHO-ONCOLOGIE or PSYCHO-ONCOLOGY or RADIATION-ONCOLOGY or RADIOLOGY-AND-ONCOLOGY or RADIOTHERAPY-AND-ONCOLOGY or RECENT-PATENTS-ON-ANTI-CANCER-DRUG-DISCOVERY or RENAISSANCE-OF-CANCER-IMMUNOTHERAPY or SEMINARS-IN-CANCER-BIOLOGY or SEMINARS-IN-ONCOLOGY or SEMINARS-IN-RADIATION-ONCOLOGY or STRAHLENTHERAPIE-UND-ONKOLOGIE or SUCCESSES-AND-LIMITATIONS-OF-TARGETED-CANCER-THERAPY or SUPPORTIVE-CARE-IN-CANCER or SURGICAL-ONCOLOGY-CLINICS-OF-NORTH-AMERICA or SURGICAL-ONCOLOGY-OXFORD or TARGETED-ONCOLOGY or TECHNOLOGY-IN-CANCER-RESEARCH-TREATMENT or THERAPEUTIC-ADVANCES-IN-MEDICAL-ONCOLOGY or THORACIC-CANCER or TRANSLATIONAL-ONCOLOGY or TUMOR-BIOLOGY or TUMORI or TUMOR-MICROENVIRONMENT-AND-CELLULAR-STRESS-SIGNALING-METABOLISM-IMAGING-AND-THERAPEUTIC-TARGETS or UHOD-ULUSLARARASI-HEMATOLOJI-ONKOLOJI-DERGISI or UROLOGIC-ONCOLOGY-SEMINARS-AND-ORIGINAL-INVESTIGATIONS or VETERINARY-AND-COMPARATIVE-ONCOLOGY or WORLD-JOURNAL-OF-SURGICAL-ONCOLOGY or WSPOLCZESNA-ONKOLOGIA-CONTEMPORARY-ONCOLOGY) or (TI=((ANTITUMOR* NOT NECROSIS) or (HPV* NOT PARVO*) or (IRRADIATION AND FRACTIONATED) or (MYC NOT (C or N)) or (PML AND (APOPTO* or GENE or NUCLEAR or NUCLEUS or PROTEIN* or RAR* or UBIQUITIN)) or (TOPOISOMERASE AND INHIBITOR) or (TUMO*R* NOT NECROSIS) or 5T4 or ADENOCARCINOMA or ADENOCARCINOMAS or ADENOMA or ADENOMAS or ADENOSARCOMAS or ADENOSARCOMA or ADRIAMYCIN or AGR3 or AKAP13 or ALEMTUZUMAB or ALEX2 or ALITRETINOIN or ALTRETAMINE or AMELOBLASTOMA or AMELOBLASTOMAS or AMIFOSTINE or AML or ANASTROZOLE or ANGIOSARCOMA or ANGIOSARCOMAS or ANTICANCER* or ANTICARCINO* or ANTILEUKEMIC or ANTIMELANOMA or ANTIMYELOMA or ANTINEOPLAS* or ANTIPROLIF* or ANTITUMO*R or ARIMIDEX* or ARMCX1 or AROMATASE or ASTROCYTOMA or ASTROCYTOMAS or AZACITIDINE or B-8801 or BCAR1 or BCL2 or BCL2 or BCR-ABL or BCR/ABL or BICALUTAMIDE or BIN2 or BIOREDUCTIVE or BLEOMYCIN or BORTEZOMIB or BRAF or BRAP1 or BRCA or BRCA1 or BRCA2 or BRCC3 or BRACHYTHERAPY or BRI3BP or BRMS1 or BRYOSTATIN* or BUSULFAN or C2ORF40 or CAELYX* or CAGE1 or CAGE-1 or CANCER* or CAPECITABINE or CARBOGEN or CARBOPLATIN or CARCINO* or CARMUSTINE or CDKN2A or CDX2 or CEA or

CEP290 or CERVICAL SMEAR or CETUXIMAB or CHEMOPREVENT* or CHEMORADIOTHERAPY or CHEMOSENSITIV* or CHEMOTHERAP* or CHLORAMBUCIL or CHOLANGIOCARCINOMA or CHOLANGIOCARCINOMAS or CHONDROSARCOMA or CHONDROSARCOMAS or CHORIOCARCINOMA or CHORIOCARCINOMAS or CIN or CISPLATIN or CLADRIBINE or CLL or CML or COMBRETASTATIN or CRANIOPHARYNGIOMA or CRANIOPHAR YNGIOMAS or CT45-1 or CT47 or CYCLOPHOSPHAMIDE or CYSTADENOCARCINOMA or CYSTADENOCARCINOMAS or CYSTADENOMAS or CYSTADENOMA or CYTARABINE or CYTOSINE-ARABINOSIDE or DACARBAZINE or DASATINIB or DAUNORUBICIN or DBC1 or DDX53 or DECITABINE or DERMATOFIBROSARCOMA or DERMATOFIBROSARCOMAS or DOCETAXEL or DOXORUBICIN* or DU-PAN-2 or DYSGERMINOMA or DYSGERMINOMAS or EBAG9 or ECRG or EEF1A1 or ELAC2 or EORTC or EPENDYMOMA or EPENDYMOMAS or EPIRUBICIN or ERBB* or ERLOTINIB or ESTRAMUSTINE or ETOPOSIDE* or ETV2 or EXEMESTANE or FIBROMA or FIBROMAS or FIBROSARCOMA or FIBROSARCOMAS or FLI1 or FLOXURIDINE or FLUOROURACIL or FOS or FULVESTRANT or GA50 or GANGLIOGLIOMA or GANGLIOGLIOMAS or GANGLIONEUROBLASTOMA or GANGLIONEUROBLASTOMAS or GEFITINIB or GEMCITABINE or GEMTUZUMAB or GERMINOMA or GERMINOMAS or GLEASON or GLEVEC* or GLIOBLASTOMA or GLIOBLASTOMAS or GLIOMA or GLIOMAS or GLIOSARCOMA or GLIOSARCOMAS or GLIVEC or GOSERELIN or HCCR1 or HEMANGIOBLASTOMA HEMANGIOBLASTOMAS or HEMANGIOENDOTHELIOMA or HEMANGIOENDOTHELIOMAS or HEMANGIOSARCOMA or HAMANGIOSARCOMAS or HEPATOBLASTOMA or HEPATOBLASTOMAS or HEPATOCARCINO* or HEPATOMA or HEPATOMAS or HER2 or HERCEPTIN* or HISTIOCYTOMA or HISTIOCYTOMAS or HODGKIN DISEASE or HODGKINS or HRPT2 or HYDROXYUREA or HYPERNEPHROMA or HYPERNEPHROMAS or IBRITUMOMAB-TIUXETAN or IDARUBICIN or IFOS*AMIDE* or IMATINIB or IMRT or INSULINOMA or INSULINOMAS or INTRATUMOR* or IODINE-131-ANTI-B1-ANTIBODY or IPILIMUMAB or IRESSA or IRINOTECAN or IXABEPILONE or JUN or L514S or L552S or LAPATINIB or LCAP or LEIOMYOMA or LEIOMYOMAS or LEIOMYOSARCOMA or LEIOMYOSARCOMAS or LENALIDOMIDE or LETMD1 or LETROZOLE or LEUKAEM* or LEUKEM* or LI FRAUMENI or LIPOSARCOMA or LIPOSARCOMAS or LOMUSTINE or LY2K or LYMPHOBLASTIC or LYMPHOMA* or LYMPHOPROLIFERATIVE or MACC1 or MALIGNANC* or MALIGNANT or MAMMOGRA* or MAP3K8 or MASTECTOM* or MEDULLOBLASTOMA or MEDULLOBLASTOMAS or MELANOMA or MELANOMAS or MELPHALAN* or MENINGIOMA or MENINGIOMAS or MERCAPTOPURINE or MESOTHELIOMA or MESOTHELIOMAS or METASTAS* or METASTAT* or METHYLGUANINE or MITOMYCIN or MITOXANTRONE or MLH1 or MLL or MSH or MSH2 or MUC1 or MYB or MYELODYSPLAS* or MYELOID or MYELOMA* or MYELOPROLIFERATIVE or MYXOFRIBOSARCOMA or MYXOFRIBOSARCOMAS or NEOPLAS* or NEPHROBLASTOMA or NEPHROBLASTOMAS or NEPHROMA or NEPHROMAS or NEURINOMA or NEURINOMAS or NEUROBLASTOMA or NEUROBLASTOMAS or NEUROFIBROSARCOMA or NEUROFIBROSARCOMAS or NEUROMA or NEUROMAS or NHL or NSCLC or NUP98 or OLIGOASTROCYTOMA or OLIGOASTROCYTOMAS or OLIGODENDROGLIOMA or OLIGODENDROGLIOMAS or ONCOGEN* or ONCOLOG* or ONCOLYTIC or ONCOPROTEIN or OSTEOSARCOMA or OSTEOSARCOMAS or OXALIPLATIN or P12INK4A or

PANITUMAB or PAP-SMEAR or PAPILLOMA or PAPILLOMAS or PAX3 or PBOV1 or PEGASPARGASE or PEMETREXED or PENTOSTATIN or PEUTZ or PHEOCHROMOCYTOMA or PHEOCHROMOCYTOMAS or PHTOTODYNAMIC-THERAP* or PLASMACYTOMA or PLASMACYTOMAS or PML/RAR* or PMS1 or POLYCYTHEMIA-RUBRA or PPHLN1 or PREDNISOLONE or PROCARBAZINE or PROSTATECTOMY or PROTOONCOGEN* or PROSTATE-SPECIFIC-ANTIGEN or PTEN or RAD51 or RADIATION-THERAPY or RADIOSENSI* or RADIOSURGERY or RADIOTHERAP* or RALTITREXED or RAS or RB1 or RCVRN or RET or RETINOBLASTOMA* or RHABDOMYOSARCOMA or RHABDOMYOSARCOMAS or RHOBTB2 or SARCOMA or SARCOMAS or SCHWANNOMA or SCHWANNOMAS or SDCCAG or SEMINOMA or SEMINOMAS or SFXN4 or SKCG-1 or SLC35C2 or SNCG or SORAFENIB or SPANXC or SRC or STEAP2 or STREPTOZOCIN or SUNITINIB or TAMOXIFEN or TARCEVA or TAXOL or TAXOTERE or TBC1D3 or TCCSG or TEMODAL or TEMOZOL*MIDE or TEMSIROLIMUS or TENIPOSIDE or TERATOMA or TERATOMAS or TFF1 or THIOGUANINE or THIOTEPA or THYMOMA or THYMOMAS or TOMOTHERAPY or TOMUDEX or TOPOTECAN or TP53 or TRASTUZUMAB or TREOSULFAN or TROVAX or TSC1 or UOEH-LC-1 or VCRP PROTOCOL or VINBLASTINE or VINCRISTINE or VINORELBINE or VWA5A or WALDENSTROM* or XAGE1A or XERODERMA-PIGMENTOSUM or ZOLEDRONIC-ACID or ABIRATERONE or ANTHRACYCLINE or ANTHRACYCLINES or ANTILEUKEMIA or AXITINIB or BLINATUMOMAB or BOSUTINIB or BRENTUXIMAB or CARFILZOMIB or CATUMAXOMAB or CEDIRANIB or CERITINIB or CHEMORADIATION or CHORDOMA or CHORDOMAS or CRIZOTINIB or CYSTECTOMY or DCIS or DINACICLIB or DOVITINIB or ENZALUTAMIDE or ERIBULIN or ESOPHAGECTOMY or ESTHESIONEUROBLASTOMA or ESTHESIONEUROBLASTOMAS or FUNGOIDES or (GIST AND GASTR*) or HCC or HNSCC or IBRUTINIB or IDELALISIB or LIPOBLASTOMA or LIPOBLASTOMAS or LYMPHADENECTOMY or LYNCH-SYNDROME or NILOTINIB or OESOPHAGECTOMY or OSTEOCHONDROMA or OSTEOCHONDROMAS or PACLITAXEL or PANCREATICODUODENECTOMY or PANCREATOBLASTOMA or PANCREATOBLASTOMAS or PANCREATODUODENECTOMY or PANITUMUMAB or PARANEOPLASTIC or PAZOPANIB or POSTMASTECTOMY or PROTON-BEAM-THERAPY or PSEUDOMYXOMA or PSEUDOMYXOMAS or REGORAFENIB or SBRT or TRAMETINIB or VEMURAFENIB or VISMODEGIB or VMAT))

DRM Software Review
